# Accuracy of glucose measurements in critically ill patients

**DOI:** 10.1186/cc9826

**Published:** 2011-03-11

**Authors:** V Claverie, L Lonjaret, B Riu-Poulenc, S Silva, T Geeraerts, O Fourcade

**Affiliations:** 1 University Paul Sabatier, Toulouse, France

## Introduction

The aim as to compare the accuracy of glucose measurements using a glucose meter analyser infingerstick, arterial blood and laboratory tests in critically ill patients and to determine factors influencing the bias between these methods.

## Methods

This prospective observational study included 75 consecutive ICU patients, corresponding to 302 pairs of measures (aged 56 ± 16 years, SOFA 8 ± 4, IGS2 56 ± 20). Findings from two different methods of glucose measurement were compared with laboratory blood glucose measurements: glucose meter analysis of capillary blood (fingerstick), and glucose meter analysis of arterial blood during the first 3 days in the ICU. Agreement between measurements was assessed using the Bland-Altman method.

## Results

**Figure 1:**
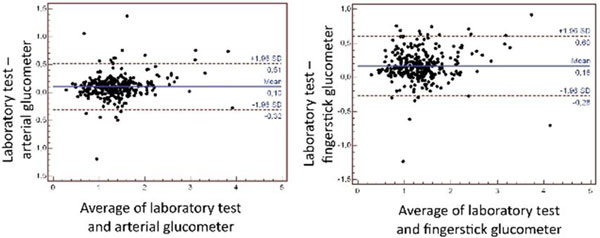


The correlation coefficient between fingerstick and laboratory results was 0.91 (95% CI = 0.89 to 0.99) and 0.92 (95% CI = 0.92 to 0.94) between arterial glucose meter analysis and laboratory testing. The mean bias between fingerstick and laboratory testing was 0.16 g/l, and between arterial glucose meter and laboratory testing was 0.10 g/l. Norepinephrine used did not affect the bias between methods (*P *= NS for all comparisons). See Figure 1.

## Conclusions

The agreement between methods for glucose level measurements appears not to be clinically acceptable. Either fingerstick or arterial glucose meter analysis have large limits of agreement with the gold standard laboratory testing for blood glucose measurements.

